# Effects of long-term continuous cropping on microbial community structure and function in tobacco rhizosphere soil

**DOI:** 10.3389/fmicb.2025.1496385

**Published:** 2025-03-14

**Authors:** Bingye Yang, Changchun Feng, Hong Jiang, Yulan Chen, Mengjiao Ding, Huaxin Dai, Zhen Zhai, Mengmeng Yang, Taibo Liang, Yanling Zhang

**Affiliations:** ^1^Zhengzhou Tobacco Research Institute of CNTC, Zhengzhou, China; ^2^Sichuan Tobacco Science Research Institute, Chengdu, China; ^3^Liangshan Branch of Sichuan Tobacco Company, Xichang, China

**Keywords:** continuous cropping, metagenomics, soil nutrients and enzymes, soil microbial community, soil microbial function

## Abstract

As is well known, continuous cropping can lead to a decrease in crop yield and quality. Despite this, continuous cropping remains prevalent in practical agricultural production, particularly in the case of tobacco cultivation, owing to its high economic value. The samples for this study were collected from a flue-cured tobacco planting base located in Huili County, Liangshan Yi Autonomous Prefecture, Sichuan Province, China. After years of continuous planting, the yield of tobacco in this base has significantly decreased. In order to explain the microecological causes of this phenomenon, we collected non-continuous cropping, continuous cropping for 5 years, and continuous cropping for 10 years of tobacco rhizosphere soil, and analyzed the effects of long-term continuous cropping on nutrients, enzyme activities, microbial community structure, and function of tobacco rhizosphere soil. The results showed that with the continuous cropping, the majority nutrients (except for phosphorus and manganese) in rhizosphere soil decreased significantly, and the rhizosphere microbial community structure changed significantly. Correlation network analysis results showed that changes in the rhizosphere microbial community of tobacco were closely related to soil urease, active organic carbon, and available iron content. The results of functional analysis based on microorganisms and genes showed that the rhizosphere microbiota may change the content of soil nutrients through iron_respiration, sulfur_respiration, and Carbon fixation in prokaryotes pathways. The results of the correlation network analysis and the functional analysis mutually confirmed each other, both emphasizing the important role of soil carbon and iron in shaping the structure of the tobacco rhizosphere microbial community. Based on the results of this study, we propose to improve the microbial community structure of tobacco rhizosphere soil by increasing the levels of readily oxidizable organic carbon, available iron, and soil urease activity in the future, so as to alleviate the negative impact of continuous cropping on crop yield. The results of this study provide theoretical support for modifying the rhizosphere microbial environment through nutrient regulation, thereby enhancing plant growth in the context of continuous tobacco cropping.

## Introduction

Tobacco, an important economic crop with a cultivation history dating back five centuries, is extensively grown worldwide (Sierro et al., 2014). Continuous cropping is a common practice in tobacco agriculture in China due to limited arable land and inappropriate farming techniques (Lei et al., 2020). Previous studies have demonstrated that prolonged continuous cropping of tobacco results in soil nutrient depletion, changes in soil enzyme activity, and alterations in soil microbial abundance, leading to an imbalance in the microecosystem (Chen et al., 2023). These factors contribute to dwarfed plants, stunted growth, reduced leaf area, increased susceptibility to diseases and pests, ultimately leading to decreased yield and quality of tobacco (Chen et al., 2023).

The soil provides the essential substrate and nutrients vital for plant growth. Suitable soil nutrient conditions play a crucial role in enhancing the yield and quality of tobacco (Zhang et al., 2016). For instance, high levels of nitrogen can stimulate excessive growth in tobacco, resulting in diminished accumulation of secondary metabolites such as nicotine, phenols, terpenes, alcohols, and lipids in tobacco (Zhai et al., 2022). Conversely, insufficient nitrogen content may lead to delayed growth in tobacco plants, characterized by dwarf, yellow, and thin. Additionally, soil also provides essential nutrients for the microorganisms residing within it. Nutrient-rich soil harbor a diverse array of microorganisms, forming a complex and resilient ecosystem that can adapt well to environmental changes and offer a stable nutrient supply for plants (Hartmann and Six, 2023). In contrast, soils with low nutrient content exhibit lower microbial diversity and richness, resulting in reduced resilience to risks (Xu Y. et al., 2023). Therefore, comprehending the impact of continuous cropping on soil nutrients is crucial for optimizing tobacco cultivation techniques and enhancing both yield and quality.

Rhizosphere soil microorganisms play a pivotal role in plant life processes. These microorganisms can facilitate plant growth and development, enhance nutrient utilization, improve plant tolerance to biological and abiotic stresses, and increase crop yields (Badri and Vivanco, 2009; Yang et al., 2009; Trivedi et al., 2020). Recent studies on various crops such as peanut (Li et al., 2014), sweet potato (Gao et al., 2019), cotton (Xi et al., 2019), and soybean (Tian et al., 2020) have highlighted the disruption of rhizosphere soil microbial community structure due to continuous cropping. Previous study on tobacco has suggested that dysfunctions or variations in the flora of soil microorganisms caused by continuous cropping leads to a reduction in the number, abundance, and diversity of probiotic bacterial populations in soil (ammonificator and nitrifier), and an increase in the number of fungi and actinomycetes (Wang et al., 2008). Continuous cropping also increases the number of pathogens in tobacco rhizosphere, exacerbating bacterial wilt disease (Niu et al., 2017). Furthermore, changes in soil microbial communities directly impact extracellular enzyme activity, which in turn affects nutrient cycling and uptake by plants (Wang et al., 2022b). Therefore, a comprehensive analysis of the effects of continuous cultivation on the diversity and function of rhizosphere microbial communities is crucial for understanding how continuous cropping influences rhizosphere microorganisms, alters soil nutrient cycling, and hinders plant growth.

Soil metagenomics technology has been widely used in the study of microbial community structure and functions, substantially advancing the development of soil microecology (Semenov, 2021). In recent years, many scholars have used soil metagenomics technology to analyze the effects of continuous cropping on the diversity of soil microorganisms and their functions in the rhizosphere zone of different plants, which has made an important contribution to reducing the negative effects caused by continuous cropping (Pang et al., 2021; Gu et al., 2022; Li et al., 2023). However, there is limited literature on the changes in the tobacco rhizosphere microbiota under continuous cropping systems using soil metagenomic technology, as well as the relationship between these changes and soil nutrients or enzyme activities. Understanding the interplay among them in continuous cropping systems is crucial for tobacco cultivation and management. In this study, we hypothesized that the decline in tobacco yield in the continuous cropping system was caused by changes in the structure and function of the rhizosphere microbial community, and that there was a significant correlation between the changes in the microbial community structure and rhizosphere nutrient content or enzyme activities. To verify this hypothesis, we collected tobacco rhizosphere soils with different continuous cropping years, and analyzed the effects of continuous cropping on soil nutrients, enzyme activities and microbial community structure in the tobacco rhizosphere. Subsequently, we further analyzed the correlation between changes in soil microbial communities and soil nutrients or enzymes to identify key factors influencing tobacco growth under continuous cropping conditions. This study aims to offer valuable insights for the continuous cultivation and management of tobacco.

## Materials and methods

### Experimental design and sample collection

The study was conducted at the flue-cured tobacco planting base in Huili County, Liangshan Yi Autonomous Prefecture, Sichuan Province, China (101°50′E, 26°30′N). This region is a subtropical monsoon climate zone with distinct dry and wet, abundant solar and thermal resources, abundant rainfall, large daily temperature difference and small annual temperature difference (Xu Y. et al., 2023). Given that the natural conditions in this region are extremely suitable for high-quality flue-cured tobacco cultivation, the phenomenon of continuous cropping is very common, resulting in the presence of tobacco planting areas with different continuous cropping years in the base. The experiment was carried out in September 2022, encompassed three distinct planting areas within the base with different cropping histories (non-continuous cropping, continuous cropping for 5 years, continuous cropping for 10 years). Five representative sample sites were selected for each planting area to collect soil samples. The collection methods of rhizosphere soil were slightly changed according to Fu et al. (2017). The tobacco plants were pulled out as a whole, and the bulk soil and the impurities attached to the tobacco root surface were removed. Subsequently, the roots were shortened to ~10 centimeters in length. The shortened roots were then placed into a 500 mL centrifuge bottle, treated with ultrasonic waves at 70 Hz for 30 min, and shaken at 28°C for an additional 30 min. The root system was removed, the soil remaining in the centrifuge bottle was stored, which was the tobacco rhizosphere soil sample. A portion of each soil sample was immediately shipped from the field to the laboratory in an ice box and immediately stored at −80°C for DNA extraction. The remaining portion of soil samples was air-dried at room temperature and stored for physicochemical analysis.

### Determination of soil nutrients and pH

The study employed various methods to analyze the soil properties: soil total organic carbon levels were determined using the high-temperature heating potassium dichromate oxidation volumetric method (Li et al., 2017), while the content of readily oxidizable organic carbon (ROC) was assessed through the potassium permanganate oxidation method (Blair et al., 1995). Soil total nitrogen was determined using the semi micro Kelvin method, available phosphorus content was measured via NaHCO_3_ extraction-molybdenum antimony colorimetric method (Wang et al., 2023), and available potassium was extracted with ammonium acetate and measured by flame photometry (Pansu and Gautheyrou, 2007). Water-soluble chlorine was determined by silver nitrate titration, water-soluble calcium and magnesium were analyzed by EDTA titration, and the content of available copper, zinc, iron, and manganese was assessed through hydrochloric acid extraction and atomic absorption spectrophotometry. The soil pH value was measured using the potentiometer method with a water-soil ratio of 4:1.

### Determination of soil enzyme activities

Soil sucrase, catalase, urease, and acid phosphatase activities were assessed using different methods: 3,5-dinitrosalicylic acid colorimetry for sucrase, potassium permanganate titration for catalase, colorimetric analysis of sodium phenate-sodium hypochlorite for urease, and p-nitrobenzene photosphate disodium colorimetric for acid phosphatase (Yu et al., 2022).

### Soil metagenome sequencing

Total genomic DNA was extracted from 15 rhizosphere siol samples (5 for each treatment) using the PowerSoil DNA isolation kit (Mo Bio Laboratories, Inc., USA) according to the manufacturer's instructions. The concentration and purification of soil DNA were estimated using a NanoDrop 2000 spectrophotometer (Thermo Scientific, Waltham, MA, United States), and DNA quality was checked by 1% agarose gel electrophoresis. Sonication was used to fragment the DNA sample to a size of about 350 bp, and then DNA fragments were purified by Agencourt AMPure XP Nucleic Acid Purification Kit (Beckman Coulter, Germany). High-quality libraries were constructed using the Next Ultra™ II DNA Library Preparation Kit (NEB, UK), and then amplified and purified the libraries with the Next Q5 Hot Start HiFi PCR Master Mix (NEB, UK). After library profile analysis using an Agilent 2100 Bioanalyzer (Agilent Technologies, USA) and qPCR quantification (MxPro, Agilent Technologies, USA), each library was sequenced using a paired-end flow cell on Illumina Miseq PE 300 high-throughput sequencer (Majorbio Corporation, Shanghai, China).

First, low-quality metagenomic reads (length < 50 bp or with a quality value < 20) were removed by fastp software (version 0.22.0). Subsequently, the reads were aligned to the host DNA sequence by the Bowtie software (version 2.3.5) and the contaminating reads with high alignment similarity were removed. The resulting data, termed clean data, were then subjected to metagenome assembly using the MEGAHIT assembly software (Version v1.2.9; Zeller et al., 2014).

The open reading frames (ORFs) from each sample were predicted using METAProdigal, and ORFs with a length ≥ 100 bp were translated to amino acid sequences. Combine ORF prediction results from all samples and mixed assembly, and use CD-HIT software (Version 4.8.1) for redundancy removal, generating the initial gene catalog consisting of non-redundant nucleic acid sequences. The abundance information of the gene catalog in each sample can be obtained by combining the clean data of each sample. Core -gene dilution curves were further analyzed to assess stability of the assayed samples (Xu Y. Q. et al., 2023). The gene catalog was functionally annotated to orthologous groups in the KEGG databases (version 62) using Smash Community (version 1.6; Sunagawa et al., 2015). Gene abundance profiles were generated by mapping high quality reads from each sample to the gene catalog.

The unique gene set was compared with those of bacteria, fungi, archaea, and viruses extracted from the NCBI NR database using DIAMOND software (Version v0.9.25). The alignment sequence with the highest similarity was taken as the species annotation information of the sequence. The abundance of the each species in every sample was calculated based on the total abundance of ORFs, and the abundance of species in each sample was counted at the taxonomic levels of domain, kingdom, phylum, class, order, family, genus, and species.

### Data analysis

The analysis process included alpha and beta diversity assessment, microbial community structure analysis, dominant microorganism identification, differential microorganism analysis, correlation network analysis, community function prediction, and gene functional prediction. FAPROTAX database and FUNGuild software were used to predict the function of bacteria and eukaryotes, while KEGG databases were employed for gene functional annotation. Statistical analyses such as Kruskal-Wilcox tests and Spearman correlation analysis were conducted using SPSS 21.0 software. Welch's *t*-test was used to calculate the significance of the difference between two treatments using STAMP software (Parks et al., 2014). The conventional bar graphs, stacked graphs, box graphs, violin graphs and gene distribution pie chart were plotted using GraphPad Prism 8. Rstudio software (R version 4.2.3) was used to produce principal component plots (R library was ggbiplot 0.55), heat maps (R library was pheatmap 1.0.12). The “corrplot” and igraph' packages in R program were used to determine the Spearman correlation and the topological attributes of the network, respectively. In order to obtain a strongly co-occurring network, we focused on the correlation which Spearman's absolute *r* > 0.50 and *P* < 0.05, then the network was visualized using “Cytoscape” software.

## Results

### The nutrients content and enzyme activities in rhizosphere soil of tobacco

**Figure 1:**
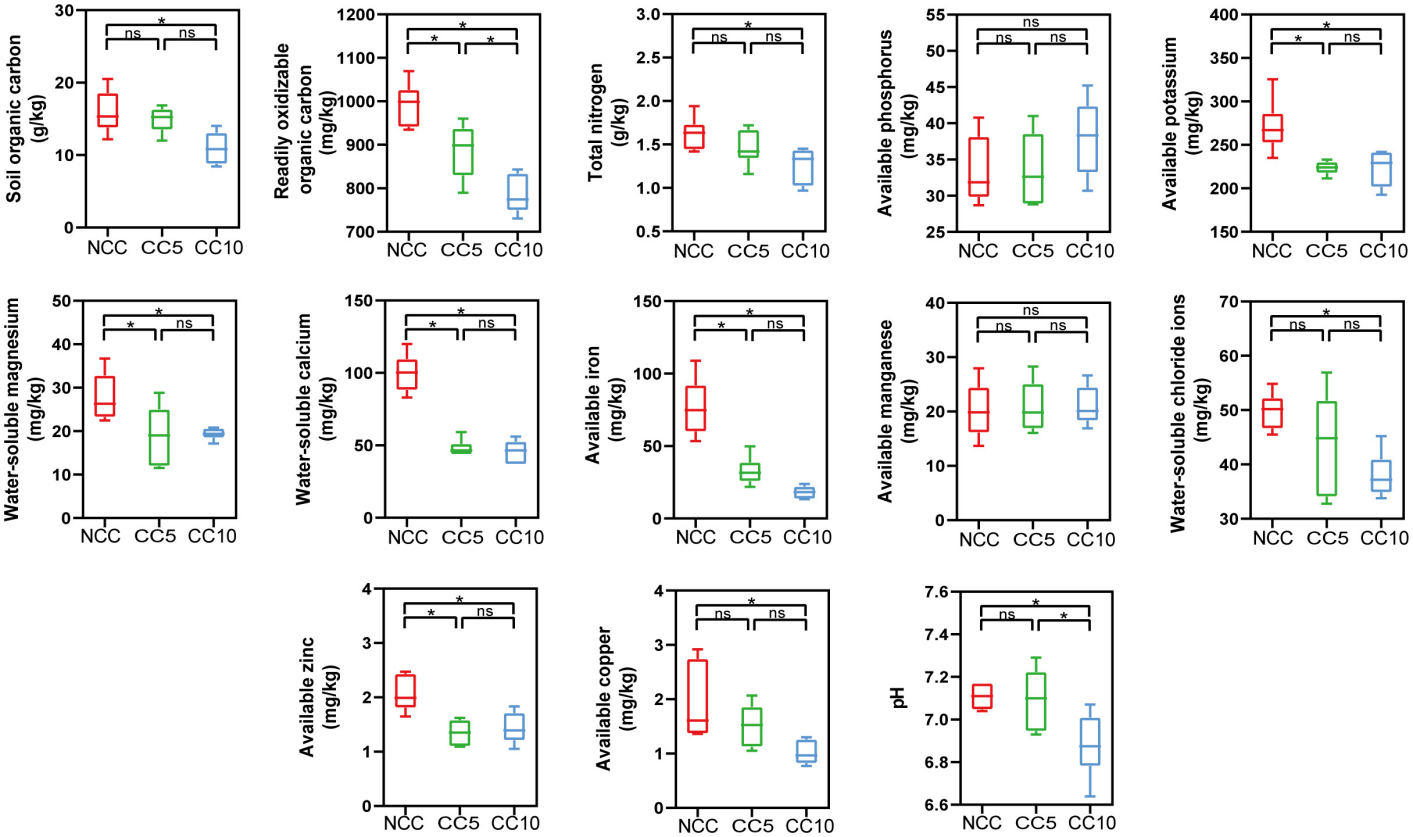
Effect of continuous cropping on the nutrients of tobacco rhizosphere soil. NCC, no continuous cropping history; CC5, 5 years of continuous cropping; CC10, 10 years of continuous cropping. The asterisk above the boxes indicate significant difference, LSD test, *P* < 0.05.

Continuous cropping has led to a decrease in tobacco yield at the tobacco planting base in Huili County. According to statistics, compared with the NCC (non-continuous cropping) soil, the yield of tobacco (Yunyan 87) planted on the CC5 (continuous planting for 5 years) soil decreased by 10.53%, and the yield of tobacco planted on the CC10 (continuous planting for 10 years) soil decreased by 22.80%. To explain the reason for the decrease in yield, the macronutrients, micronutrients, and extracellular enzyme activities in tobacco rhizosphere soils under continuous cropping for 0, 5, and 10 years were measured. The analysis of macronutrients in tobacco rhizosphere soil showed that the available phosphorus content increased from 33.44 to 38.01 mg/kg with increasing years of continuous cropping, but this increase was not significant (Figure 1). The content of other measured macronutrients decreased over time, with organic carbon content significantly decreasing from 15.93 to 10.96 g/kg, total nitrogen content significantly decreasing from 1.62 to 1.26 g/kg, and available potassium content significantly decreasing from 271.10 to 223.17 mg/kg (*p* < 0.05) (Figure 1). No significant changes in available manganese content among micronutrients, but the contents of other micronutrients significant decreased as the years of continuous cropping increased (Figure 1). For example, the available zinc and copper contents decreased significantly from 2.06 and 1.93 mg/kg in soils of NCC to 1.43 and 1.02 mg/kg in soils of CC10, respectively (*P* < 0.05; Figure 1).

**Figure 2:**
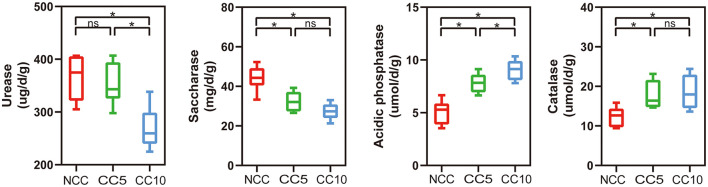
Effect of continuous cropping on the enzyme activities of tobacco rhizosphere soil. NCC, no continuous cropping history; CC5, 5 years of continuous cropping; CC10, 10 years of continuous cropping. The asterisk above the boxes indicate significant difference, LSD test, *P* < 0.05.

Furthermore, soil enzyme activity analysis showed that continuous cropping significantly increased the activities of catalase and acid phosphatase. The activity of catalase significantly increased from 12.37 μmol/d/g in NCC soil to 18.52 μmol/d/g in CC10 soil, while the activity of acid phosphatase significantly increased from 5.07 μmol/d/g in NCC soil to 9.06 μmol/d/g in CC10 soil (Figure 2). In contrast, the activities of urease and sucrase in NCC soil were significantly higher than those in continuous cropping soil. The urease activity was 365.58 μg/d/g in NCC soil, but decreased to 268.81 μg/d/g in continuous cropping soil for 10 years; The sucrose enzyme activity was 44.21 mg/d/g in NCC soil, but decreased to 27.35 mg/d/g in continuous cropping soil for 10 years (*P* < 0.05; Figure 2).

### Alpha and beta diversity of the microbial community

**Figure 3:**
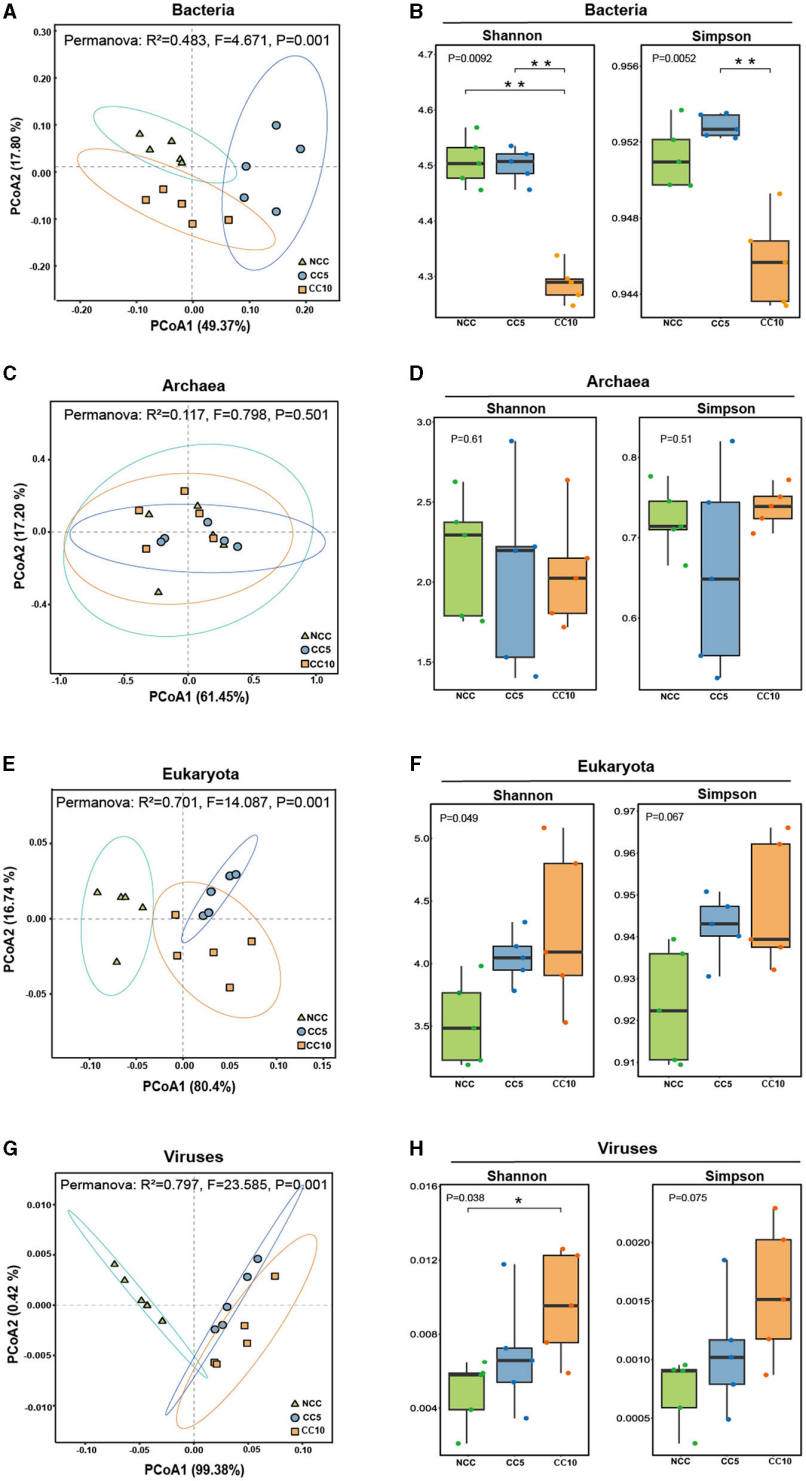
Alpha and beta diversity analysis of microbial communities in tobacco rhizosphere soil. **(A)** UniFrac-weighted PCoA plots of bacterial communities at genus level in three groups of soil samples; **(B)** Analysis of α-diversity index of bacterial in the tobacco rhizosphere soil; **(C)** UniFrac-weighted PCoA plots of archaea communities at genus level in three groups of soil samples; **(D)** Analysis of α-diversity index of archaea in the tobacco rhizosphere soil; **(E)** UniFrac-weighted PCoA plots of eukaryota communities at genus level in three groups of soil samples; **(F)** Analysis of α-diversity index of eukaryota in the tobacco rhizosphere soil; **(G)** UniFrac-weighted PCoA plots of viral communities at genus level in three groups of soil samples; **(H)** Analysis of α-diversity index of viral in the tobacco rhizosphere soil. Shannon index was conducted to characterize the evenness within groups and analyzed in Kruskal-Wallis to test for differences. Simpson index was conducted to characterize the richness within groups and analyzed in Kruskal-Wallis to test for differences. The asterisk above the bars indicate significant difference between the two, Student's *t*-test, *P* < 0.05. NCC, no continuous cropping history; CC5, 5 years of continuous cropping; CC10, 10 years of continuous cropping.

The β-diversity analysis of rhizosphere soil microorganisms with different continuous cropping years showed that bacteria, eukaryota, and viruses in the samples could be effectively distinguished according to the planting years (Figures 3A, E, G). However, archaea in samples could not be effectively distinguished based on the planting years (Figure 3C). This indicated that there were significant differences in bacteria, eukaryota, and viruses among the three groups of soil samples, and it was meaningful to classify the soils according to the continuous cropping years.

The α-diversity analysis showed that the shannon index of bacterial communities was 4.5074 in NCC, 4.5206 in CC5, and significantly decreased to 4.3089 in CC10 (Figure 3B). The Simpson index was 0.9513 in NCC, slightly increased to 0.9528 in CC5, and significantly decreased to 0.9457 in CC10 (Figure 3B). These findings indicated that the bacterial richness and evenness of rhizosphere soil decreased first and then increased, and the diversity of CC10 was significantly lower than that of NCC and CC5. For eukaryotic communities, the Shannon index increased from 3.5310 in NCC to 4.2830 in CC10, and the Simpson index increased from 0.9236 in NCC to 0.9475 in CC10 (Figure 3F). The Shannon index of the viral community increased significantly from 0.0048 in NCC to 0.0096 in CC10, and the Simpson index increased from 0.0007 in NCC to 0.0015 in CC10 (Figure 3H). These results showed that the richness and evenness of eukaryotic and viral communities increased with the duration of continuous cropping years, but there was no significant difference in the α-diversity of archaea among the three groups (Figure 3D).

### Changes in the microbial community structure of rhizosphere soil

**Figure 4:**
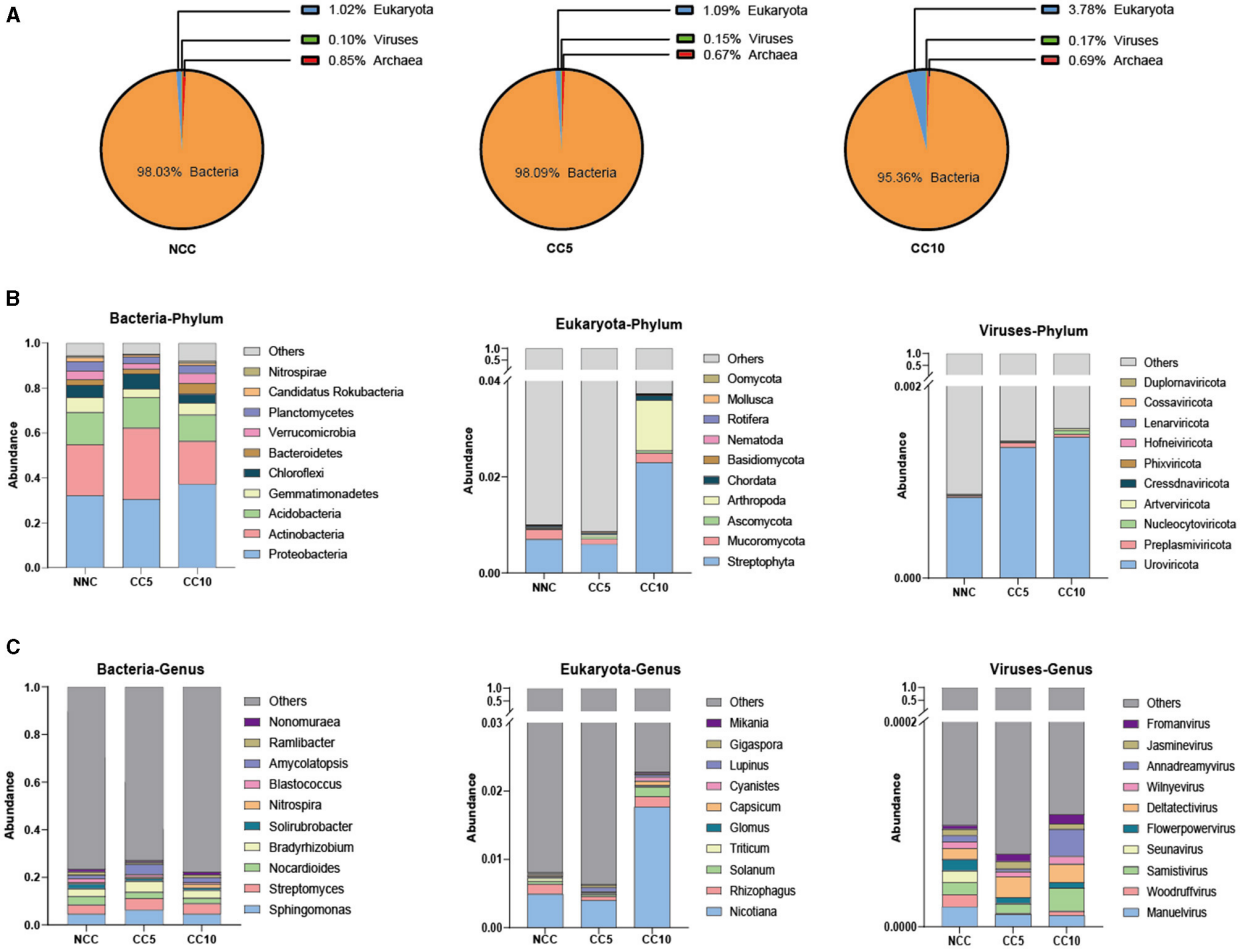
Analysis of microbial community structure in tobacco rhizosphere soil. **(A)** Abundance analysis of soil microorganisms at kingdom level; **(B)** Abundance analysis of major phyla in bacteria, eukaryota and viruses kingdoms in different continuous cropping years; **(C)** Abundance analysis of the major genus in bacteria, eukaryota and viruses kingdoms in different continuous cropping years. NCC, no continuous cropping history; CC5, 5 years of continuous cropping; CC10, 10 years of continuous cropping.

Overall, bacteria accounted for the vast majority of tobacco rhizosphere soil microorganisms, which were 98.03% (NCC), 98.09% (CC5), and 95.36% (CC10), respectively. The proportion of fungi among tobacco rhizosphere soil microorganisms increased with the duration of continuous cropping years (Figure 4A), and the proportion of fungi in CC10 soil was significantly higher than that in NCC and CC5 (*P* < 0.05, LSD).

On this basis, the microbial community structure of rhizosphere soil under continuous tobacco planting was further analyzed. In terms of bacterial phylum classification, the top five dominant microorganisms in tobacco rhizosphere soil of NCC and CC5 were Proteobacteria, Actinobacteria, Acidobacteria, Gemmatimonadetes, and Chloroflexi, while the top five dominant microorganisms in tobacco rhizosphere soil of CC10 were Proteobacteria, Actinobacteria, Acidobacteria, Gemmatimonadetes, and Bacteroidete (Figure 4B). In terms of phylum classification of viruses, the top five dominant phyla in the rhizosphere soil of tobacco plants in NCC were Uroviricota, Nucleocytoviricota, Preplasmiviricota, Artverviricota, and Cressdnaviricota, while the top five main microorganisms in the other two groups with continuous cropping were Uroviricota, Nucleocytoviricota, Preplasmiviricota, Artverviricota, and Phixviricota (Figure 4B). In terms of phylum classification of eukaryota, the top five dominant microorganisms among different continuous cropping years were the same, which are Streptophytota, Arthropoda, Mucoromycota, Chordata, and Ascomycota (Figure 4B). At the genus level, the top five dominant bacteria in tobacco rhizosphere soil in the NCC group were *Sphingomonas, Streptomyces, Nocardioides, Bradyrhizobium*, and *Solirubrobacter*, while the top five dominant microorganisms in the rhizosphere soil of the CC5 and CC10 groups were *Sphingomonas, Streptomyces, Bradyrhizobium, Amycolatopsis*, and *Nocardioide*s (Figure 4C). The top five dominant genera of eukaryota in tobacco rhizosphere soil in the NCC group were *Nicotiana, Rhizophagus, Solanum, Glomus*, and *Triticum*, the top five dominant genera in CC5 group were *Nicotiana, Rhizophagus, Solanum, Lupinus*, and *Gigaspora*, while the top five main genera in the CC10 group were *Nicotiana, Rhizophagus, Solanum, Capsicum*, and *Cyanistes* (Figure 4C). The top five dominant genera of viruses in the rhizosphere soil of NCC group were *Manuelvirus, Samistivirus, Woodruffvirus, Seunavirus*, and *Flowerpowervirus*, the top five dominant genera in the rhizosphere soil of CC5 group were *Manuelvirus, Samistivirus, Deltatectivirus, Fromanvirus*, and *Jasminevirus*, while the top five main genera in the rhizosphere soil of CC10 group were *Manuelvirus, Samistivirus, Deltatectivirus, Fromanvirus*, and *Annadreamyvirus* (Figure 4C). It can be seen that at the phylum level, there was a difference in the dominant rhizosphere soil bacteria between the CC10 group and the other two groups, and there was a difference in the rhizosphere dominant viruses between the NCC group and the other two groups; At the genus level, there was a difference in the dominant rhizosphere soil bacteria between the NCC group and the other two groups, and there were differences in the dominant eukaryota and viruses in the rhizosphere soil among the three groups.

### Screening and classification analysis of differential microorganisms

**Figure 5:**
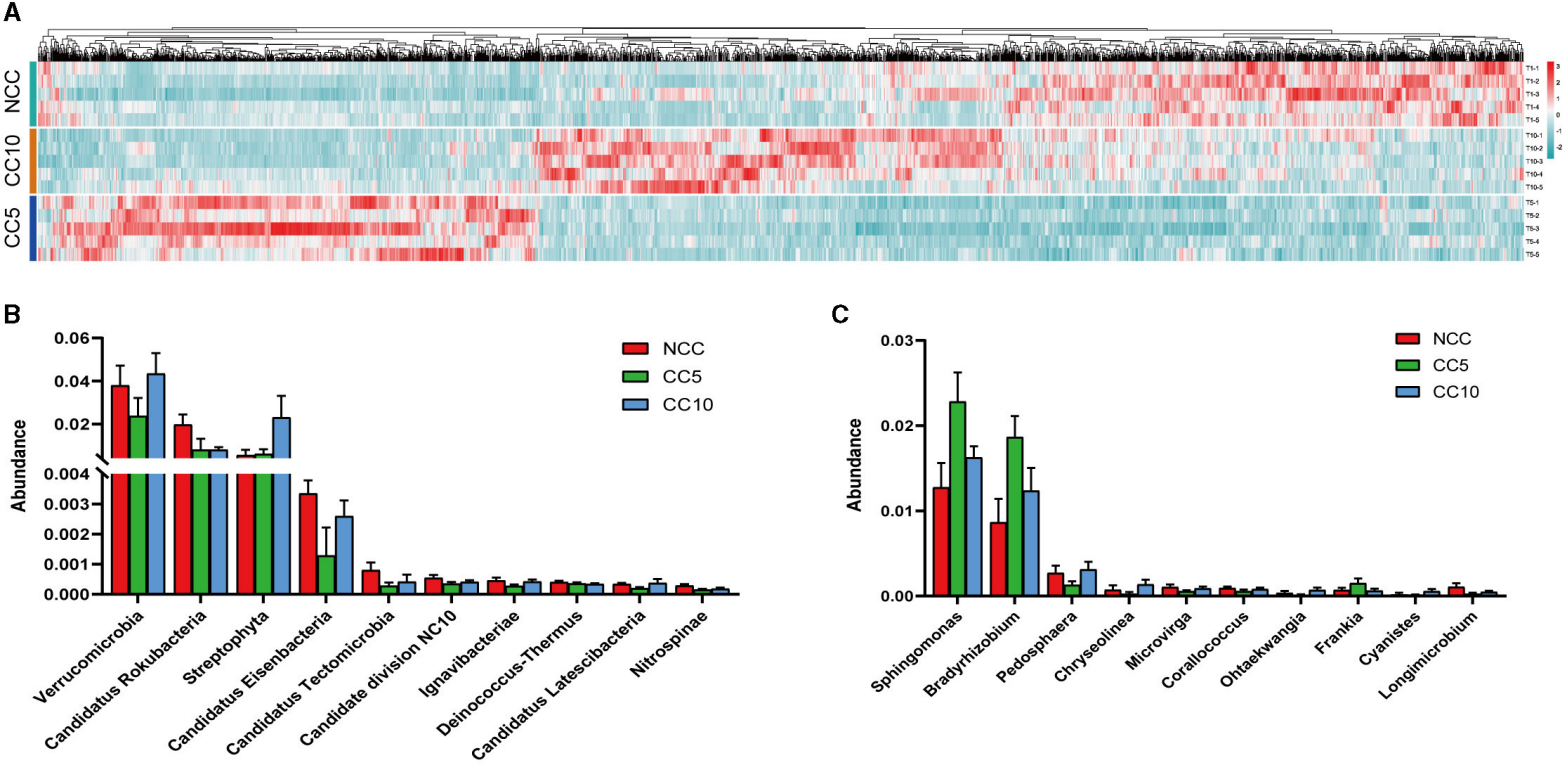
Screening and analysis of differential soil microorganisms. **(A)** Tukey-Kramer test to obtain a heat map of 2,357 microbes with significant differences between groups; **(B)** Column diagram of the top 10 differential microorganisms in terms of abundance at the phylum level; **(C)** Column diagram of the top 10 differential microorganisms in terms of abundance at the genus level. NCC, no continuous cropping history; CC5, 5 years of continuous cropping; CC10, 10 years of continuous cropping.

Following the initial analysis, this study further analyzed differences in microbial abundance among the three groups of samples (NCC, CC5, and CC10) and a total of 2,357 microbial species exhibiting significant differences were obtained, including 71 archaea and 23 viruses (Tukey-Kramer, *P* < 0.05). Heat map analysis revealed that among the 2,357 microorganisms, 856 microorganisms were obviously concentrated in the NCC group, 737 microorganisms were obviously concentrated in the CC5 group, and 764 microorganisms were obviously concentrated in the CC10 group (Figure 5A). This result showed that there were significant differences in the microbial abundance of tobacco rhizosphere soil with different continuous cropping years. At the phylum level, the top 10 phyla with the highest relative abundance of differential microorganisms in the rhizosphere soil of tobacco across different continuous cropping years were mainly Verrucomicrobia, Candidatus Rokubacteria, Streptophyta, Candidatus Eisenbacteria, Candidatus Tectomicrobia, Candidate division NC10, Ignavibacteriae, Deinococcus-Thermus, Candidatus Latescibacteria, and Nitrospinae (Figure 5B). At the genus level, the top 10 genera with the highest relative abundance of differential microorganisms in the rhizosphere soil of tobacco with varying continuous cropping years were mainly *Sphingomonas, Bradyrhizobium, Pedosphaera, Chryseolinea, Microvirga, Corallococcus, Ohtaekwangia, Frankia, Cyanistes*, and *Longimicrobium* (Figure 5C).

### Correlation network analysis between differential microorganisms and soil properties

**Figure 6:**
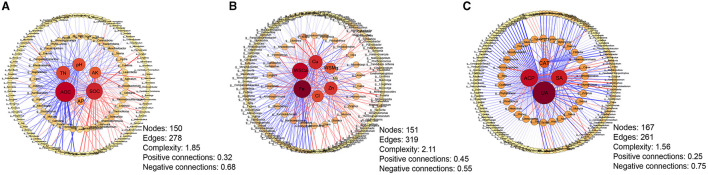
The correlation network between soil properties and differential genera among different cropping years. **(A)** Correlation network between soil macronutrients and differential genera; **(B)** Correlation network between soil micronutrients and differential genera; **(C)** Correlation network between soil enzyme activities and differential genera. Red lines indicate positive correlations, while blue lines indicate negative correlations. The thickness of each edge is proportional to the value of the Spearman correlation. The size and color of nodes indicate the number of edges connected to each node. AP, available phosphorus; SOC, soil organic carbon; ROC, readily oxidizable organic carbon; TN, total nitrogen; AK, available potassium; Cu, available copper; Fe, available iron; Mn, available manganese; Zn, available zinc; Cl, water-soluble chlorideions; WSCa, water-soluble calcium; WSMg, water-soluble magnesium; CAT, catalase; ACP, acidic phosphatase; SA, saccharase; UA, urease.

The correlation network analysis between differential genera and soil properties was carried out to further explore the relationship between microbial changes and soil properties under different continuous cropping years (Figure 6). For macroelements, the strongest correlation was observed between ROC content and differential microorganisms (degree = 82, betweenness centrality = 0.47313), whereas the weakest correlation was found between available phosphorus content and these microorganisms (Figure 6A). Among the micronutrients, the correlation between available iron content and differential microorganisms was the closest (degree = 76, betweenness centrality = 0.32627), while the correlation between available manganese content and differential microorganisms was the weakest (Figure 6B). In terms of soil enzyme activities, urease showed the strongest association with differential microorganisms (degree = 108, betweenness centrality = 0.64500), whereas catalase exhibited the weakest correlation (Figure 6C). These results indicated that soil urease, ROC, and available iron content play significant roles in influencing the changes of tobacco rhizosphere microorganisms.

### Functional analysis based on rhizosphere microorganisms

**Figure 7:**
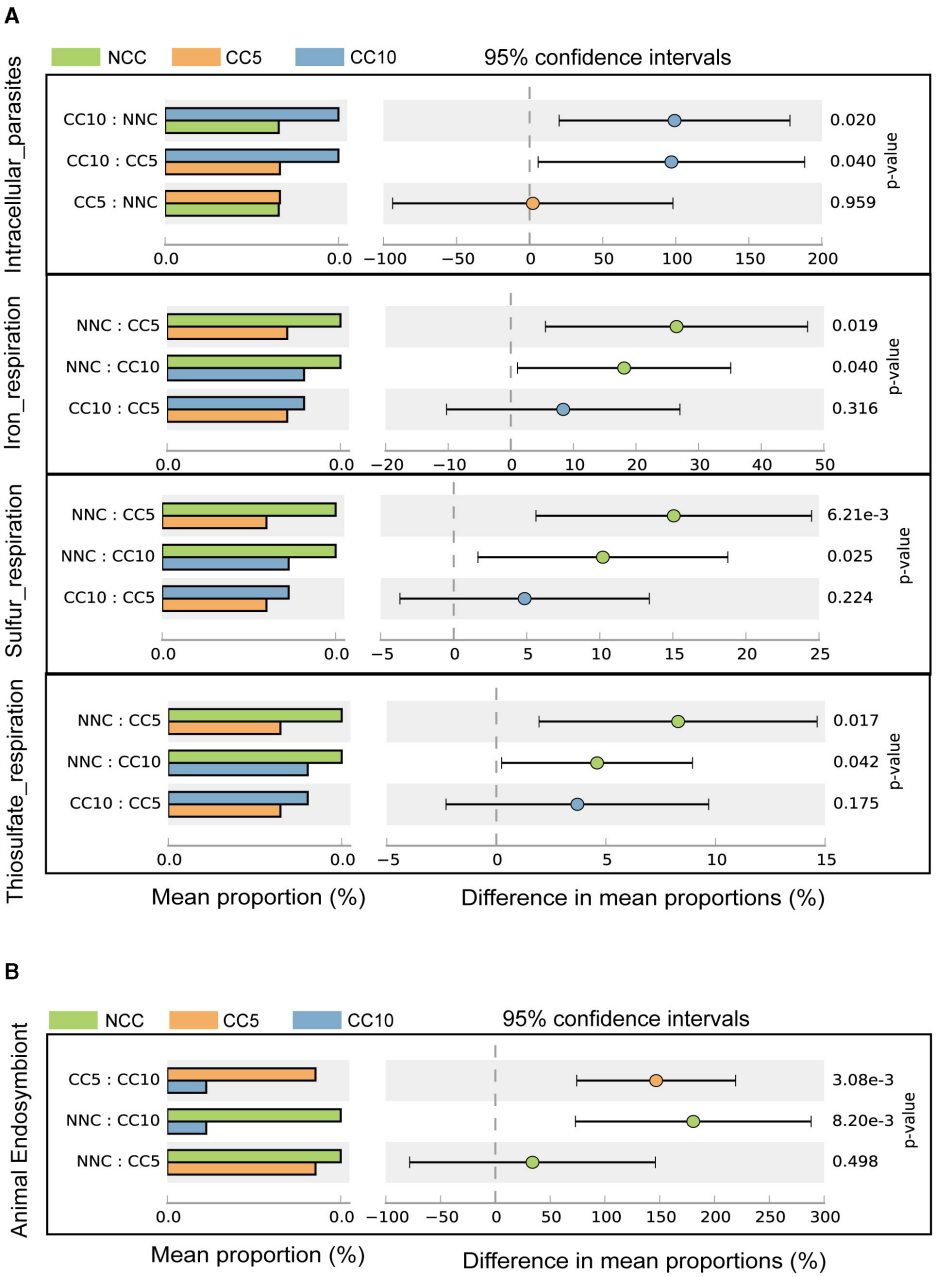
Function analysis based on microorganisms. **(A)** Difference in prokaryotic microorganisms function in tobacco rhizosphere soil samples with different continuous cropping years (Welch's *t*-test; *P* < 0.05); **(B)** Difference in eukaryotes microorganisms function in tobacco rhizosphere soil samples with different continuous cropping years (Welch's *t*-test; *P* < 0.05). NCC, no continuous cropping history; CC5, 5 years of continuous cropping; CC10, 10 years of continuous cropping.

In the FAPROTAX database, which is used for predicting microbial ecological functions, annotated bacterial genera were categorized into 69 predicted functional groups. The results of Welch's *t*-test analysis showed that there were significant differences in four functional groups, which were intracellular_parasites, iron_respiration, sulfur_respiration, and thiosulfate_respiration (Figure 7A). Bacteria related to the function of intracellular_parasites were gradually enriched in the tobacco rhizosphere with the increase of continuous cropping years, while bacteria related to iron_respiration, sulfur_respiration, and thiosulfate_respiration functions decreased gradually with the increase of continuous cropping years (Figure 7A). In addition, annotated eukaryotes were assigned to 34 predicted functional groups based on FUNGuild software. The Welch's *t*-test analysis indicated that only animal endosymbiont exhibited significant differences among the 34 functional groups, and the eukaryotes related to this function in the tobacco rhizosphere gradually decreased with the increase of continuous cropping years (Figure 7B).

### Functional analysis based on genes

**Figure 8:**
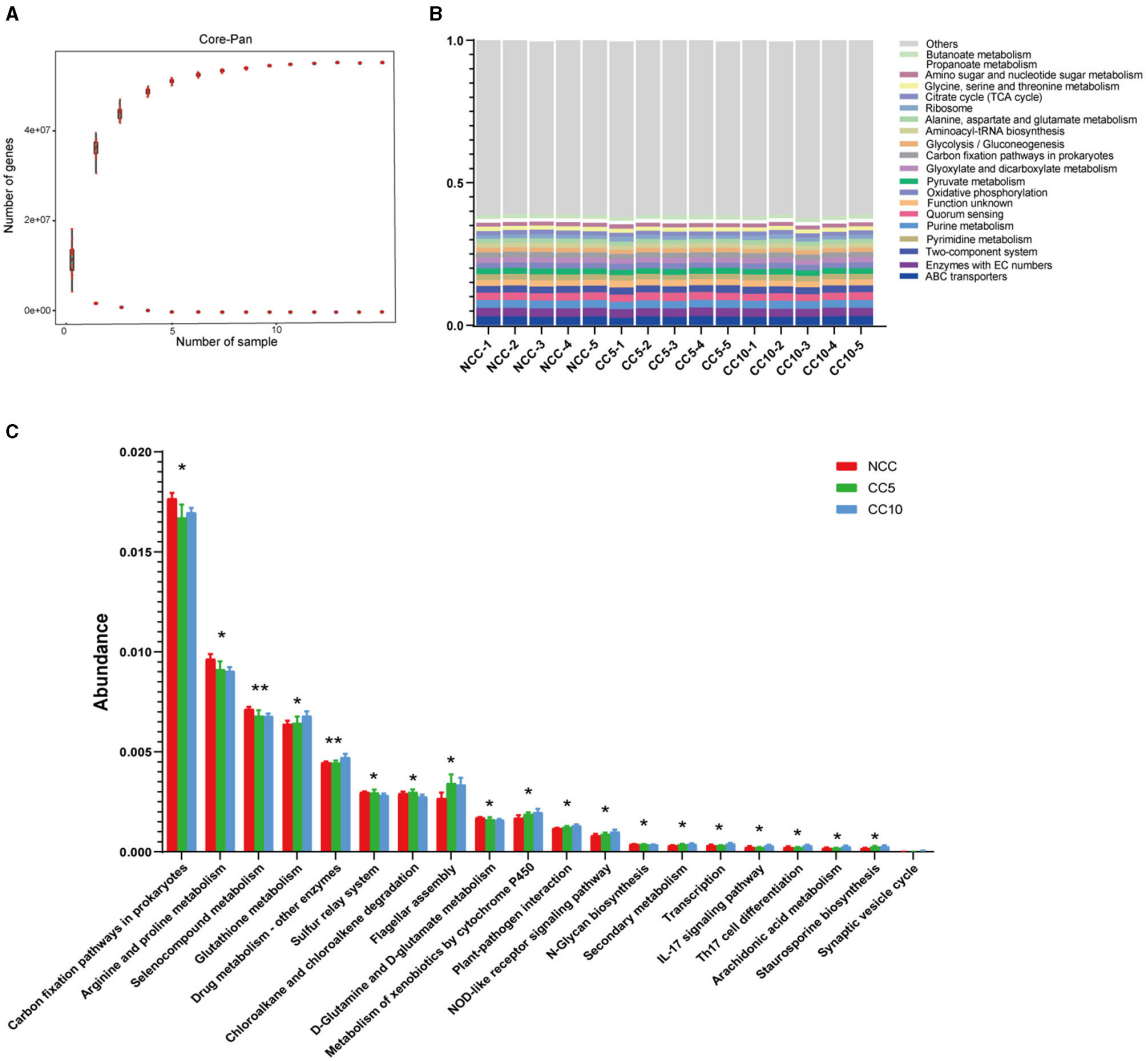
Function analysis based on genes. **(A)** Core gene dilution curve of tobacco rhizosphere soil; **(B)** Annotation of genes in level 3 KEGG pathways under different continuous cropping years; **(C)** Level 3 KEGG pathways with significant differences under different continuous cropping years (kruskal_wilcox test, *P* < 0.05). NCC, no continuous cropping history; CC5, 5 years of continuous cropping; CC10, 10 years of continuous cropping.

The rhizosphere soil of tobacco subjected to varying durations of continuous cropping was analyzed using Illumina sequencing, resulting in a total of 1,489,658,658 reads collected from fifteen samples. Open Reading Frame (ORF) prediction was conducted, yielding a total of 14,975,385 genes. The combined length of these genes in the gene catalog was 8,387.5 Mbp, with an average gene length of 560.09 bp. Analysis of the dilution curve of the Core-Pan genes indicated that core genes decreased with the increase in sample size, while the pan genes increased with the sample size (Figure 8A). Notably, when the sample size reached nine, the number of core genes and Pan tended to be stable. It can be seen that the metagenomic sequencing results of soil samples in this study effectively represent the vast majority of genes present in the soil, and the sequencing results can be utilized for further gene function analysis (Figure 8A). KEGG pathway annotation analysis of these genes showed that the most significant enriched pathway was ABC transporter, followed by enzymes with EC numbers, purine metabolism, quorum sensing, and the two-component system (Figure 8B). Subsequently, kruskal_wilcox test was used for difference analysis, and the results showed significant differences among the three groups of soil samples in 18 KEGG pathways and extremely significant differences in two KEGG pathways (Figure 8C). Among them, carbon fixation pathways in prokaryotes and sulfur relay system were associated with soil nutrient metabolism (Figure 8C). In addition, we also noticed that there were significant differences in the plant-pathogen interaction pathway among the three groups of samples, and the related genes increased with the progression of continuous cropping years (Figure 8C).

## Discussion

Long-term monoculture of plants can lead to a loss or imbalance of soil nutrients, which may negatively impact plant yield and quality (Guo et al., 2022; Wang et al., 2022a). Our research findings were consistent with this view and further revealed the complex effects of tobacco continuous cropping on soil nutrient dynamics. Previous study showed that the contents of organic carbon, nitrate nitrogen and available potassium in tobacco continuous cropping soil were significantly lower than those in non-continuous cropping soil, while the total nitrogen and available phosphorus contents were accumulated with the increase of continuous cropping years (Wang et al., 2020). Bai et al. (2019) discovered that soil pH decreased in the continuous cropping field of tobacco. Our findings in this study were consistent with previous studies on the trends of organic carbon, ROC, available phosphorus, available potassium and pH. However, our study also found that the trend in total nitrogen was inconsistent with that of Wang et al. (2020), which may be related to differences in soil types, climatic conditions, or management measures. In terms of micronutrients, Chen and his colleagues found that soil iron content decreased significantly after continuous cropping, while manganese and zinc content remained stable (Chen et al., 2018). Unlike the study by Chen et al. (2018), our results revealed that, with the exception of available manganese, the levels of other micronutrients decreased as the years of continuous tobacco planting increased. The discrepancy in findings may be related to the initial nutrient status of the soil, the length of continuous cropping years, and the nutrient absorption efficiency of crops. The decrease in elements such as iron and zinc may reflect the strong absorption of these micronutrients by tobacco, while the stability of manganese may be related to its mobility in soil. These differences emphasize the complexity of soil nutrient dynamics, as well as potential differences in the results of studies under different environmental conditions. Overall, continuous cropping led to a significant decrease in the content of most nutrients, and only two soil nutrients did not change significantly. Previous research has indicated that bacteria and fungi differ in their resource preferences and nutrient acquisition strategies, with bacteria preferring resources that are easy to decompose compared to fungi (Six et al., 2006). Therefore, changes in rhizosphere nutrient content may lead to alterations in microbial community composition.

Soil enzyme activity plays an important role in nutrient cycling, reflecting total soil biological activity. Previous studies have shown that continuous cropping can lead to changes in soil enzyme activity. For example, Wang and colleagues found that tobacco continuous cropping significantly reduced the activities of urease and catalase in soil (Wang et al., 2020), which is consistent with the trend we observed in urease activity. However, our study found that catalase activity increased under continuous cropping conditions, a difference that may be related to changes in total nitrogen content and microbial abundance in the soil (Saiya-Cork et al., 2002; Chabot et al., 2020). In addition, we found that continuous cropping significantly reduced sucrase activity but increased acid phosphatase activity. The decrease in sucrase activity may be associated with the reduction of organic matter content and carbon cycling rate in soil (Li et al., 2022). The increase in acid phosphatase activity is beneficial for promoting soil phosphorus cycling and increasing soil available phosphorus content (Magadlela et al., 2023), which aligns with the detection results of soil available phosphorus content. These results indicate that changes in soil enzyme activity were the result of plant-microbe-soil nutrient interactions, which provided an important reference for further understanding and improving soil quality.

Long-term continuous cropping may significantly change the structure and diversity of soil microbial communities. The results of PCoA analysis showed that different continuous cropping years formed significantly different microbiomes, which is consistent with the previous studies using amplicon sequencing technology (She et al., 2017; Wang et al., 2020). The analysis of the difference microorganisms in the three groups of soil samples showed that 856 microorganisms significantly differentiated NCC, 737 microorganisms significantly differentiated CC5, and 764 microorganisms significantly differentiated CC10, highlighting the impact of planting years on the composition and abundance of microbial communities in tobacco rhizosphere soil. These results collectively demonstrate that there were significant differences in the species and abundance of microorganisms in the rhizosphere soil of tobacco with different continuous cropping years, and different rhizosphere microbial environments were formed, which may have cascading effects on soil health and plant productivity. Additionally, the results of α-diversity analysis showed that continuous cropping led to a decrease in bacterial diversity and an increase in fungal and viral diversity. This phenomenon may be due to the fact that environment is selective for microorganisms and stronger selection often leads to a new cohort of microbiota that adapt to the environment (Meola et al., 2014).

A previous study has shown that long-term continuous cropping leads to the transformation of the soil microbial community structure from “bacterial type” to “fungal type” (Bai et al., 2019). Consistent with this finding, our results indicated that the proportion of fungi among tobacco rhizosphere soil microorganisms increased with the duration of continuous cropping years. Although this is consistent with previous research findings, the potential mechanisms driving this transformation are worth further exploration. For instance, is the increase in fungal proportion related to factors other than continuous cropping years? Understanding all the factors driving this transformation is crucial for improving the long-term impact of continuous cropping on soil microbial species. Our analysis of dominant species at the phylum level showed that the relative abundance of Chloroflexi in NCC group was higher than that in the continuous cropping group, while the relative abundance of Bacteroides in the continuous cropping group was higher than that in the NCC group. Members of the phylum Chloroflexi are capable of fixing inorganic CO_2_ (Gaisin et al., 2017; Keppen et al., 2000) and also aerobically oxidize carbon monoxide (Islam et al., 2019; King and King, 2014). The ability of Chloroflexi phylum members was supported by our gene-based functional analysis, which showed that genes related to carbon fixation pathways in prokaryotes were significantly more abundant in NCC than in continuous cropping soils. Therefore, we speculated that the rhizosphere microorganisms in the NCC group could better fix carbon, and soil carbon content has a positive impact on crop yield. The analysis of dominant species at the genus level showed that the relative abundance of *Solirubrobacter* in the NCC group was higher than that in the continuous cropping group, while the relative abundance of *Amycolatropis* in the continuous cropping group was higher than that in the NCC group. *Solirubrobacter* has been reported to be associated with resistance to biotic and abiotic stresses (Wu et al., 2024; Otlewska et al., 2017) and has a positive effect on plant growth (Franke-Whittle et al., 2015). Furthermore, gene-based functional analysis showed that the abundance of genes related to plant-pathogen interaction pathways was significantly higher in the rhizosphere soil of continuous cropping group. Therefore, we speculated that tobacco plants in the non-continuous cropping group had better tolerance to biotic stresses. In summary, continuous cropping changed the structure and function of tobacco rhizosphere microbial communities, and the rhizosphere microorganisms in the NCC group had a positive effect on plant growth from the perspectives of dominant microorganisms and gene-based function analysis, which was consistent with the first half of our hypothesis.

The changes in rhizosphere microbial community structure are closely related to soil properties (Barberán et al., 2012; Lupatini et al., 2014). In recent years, network analysis has been increasingly used to explore the potential interactions in soil ecosystems (de Menezes et al., 2015; Eiler et al., 2012). In this study, we employed network analysis to visualize and quantify the interactions patterns among soil properties and rhizosphere microorganisms under continuous cropping systems. The results showed that soil urease, ROC, and available iron had a great influence on the changes of tobacco rhizosphere microorganisms, which was consistent with the latter half of our hypothesis. Soil enzymes are important biocatalysts involved in the decomposition, turnover, and mineralization of organic matter, thereby accelerating the soil nutrient cycling process (Shen et al., 2019; Zhang M. et al., 2021). The function of soil urease is to convert urea into ammonia that can be used by plants (Kumar and Garkoti, 2022). Lei et al. suggest that soil urease is the main factor affecting the composition of soil bacterial and fungal species (Lei et al., 2023), aligning with the findings of this study. ROC, a key component of the soil organic carbon pool, plays a vital role in soil nutrient mineralization, cycling, and energy release, which has fast migration, poor stability, easy oxidation, easy mineralization, and easy utilization by plants and soil microorganisms (Franzluebbers et al., 2001). Previous studies have shown that the assembly of soil microbial communities is determined by deterministic processes that are significantly influenced by ROC (Wang et al., 2017; Zhang Z. M. et al., 2021), which support our research findings on tobacco. Iron (Fe), regarded as the most abundant redox-active metal element in the Earth's crust, is involved in a biogeochemical cycle that includes Fe(III) reduction and Fe(II) oxidation. Previous studies have shown that iron and copper in micronutrients can better explain changes in microbial community structure than constant nutrients, and their impact on fungi and protists is greater than that on bacteria (Peng et al., 2022). This study suggests that iron plays an important role in adjusting the structure of rhizosphere microbial communities. In addition, functional analysis based on microbial species showed that the abundance of bacteria associated with iron-respiration gradually decreased with the increase of continuous cropping years. Iron respiration, also known as dissimilatory iron reduction, refers to the process in which microorganisms use extracellular insoluble iron oxides as terminal electron acceptors, reduce them by oxidizing electron donors coupled with Fe(III), and obtain energy from it. This process can convert Fe(III) in the soil, which cannot be utilized by plants, into Fe(II) which can be absorbed by roots. Therefore, we speculated that the decrease in the number of bacteria related to iron respiration leads to a decrease of Fe(II) content in rhizosphere, ultimately impacting plant yield and quality.

## Conclusion

Tobacco continuous cropping significantly reduced the content of most nutrients (except phosphorus and manganese) in rhizosphere soils, while altering soil enzyme activities. Continuous cropping significantly changed the structure and function of the rhizosphere microbial community. At the phylum level, the higher abundance of Chloroflexi in the NCC group may be related to its carbon fixation capability, while at the genus level, the higher abundance of *Solirubrobacter* in the NCC group may enhance resistance to biotic and abiotic stresses. Functional analysis further showed that the NCC group had a greater number of microorganisms involved in prokaryotic carbon fixation and fewer microorganisms associated with plant-pathogen interaction pathway. These findings provide theoretical support for understanding the yield decline caused by continuous cropping. Correlation network analysis revealed that soil urease, ROC and available iron were the key factors driving the changes of tobacco rhizosphere microbial community, which provided a theoretical basis for improving soil quality by regulating soil biochemical characteristics. Taken together, this study provides important references for improving soil quality and agricultural sustainability in the context of tobacco continuous cropping.

## Data Availability

The datasets presented in this study can be found in online repositories. The names of the repository/repositories and accession number(s) can be found below: https://www.ncbi.nlm.nih.gov/, PRJNA1110363.
